# Maize Kernel Batch Counting System Based on YOLOv8-ByteTrack

**DOI:** 10.3390/s25175584

**Published:** 2025-09-07

**Authors:** Ran Li, Qiming Liu, Miao Wang, Yuchen Su, Chen Li, Mingxiong Ou, Lu Liu

**Affiliations:** 1 School of Engineering, Anhui Agricultural University, Hefei 230036, China; ranmaxli@stu.ahau.edu.cn (R.L.); liuqiming@stu.ahau.edu.cn (Q.L.); shmilywm@stu.ahau.edu.cn (M.W.); yuchensu@stu.ahau.edu.cn (Y.S.); lichen111@stu.ahau.edu.cn (C.L.); 2 High-Tech Key Laboratory of Agricultural Equipment and Intelligence of Jiangsu Province, Jiangsu University, Zhenjiang 212013, China; myomx@ujs.edu.cn; 3 Wandong Comprehensive Experimental Station, Anhui Agricultural University, Chuzhou 239400, China

**Keywords:** seed count, YOLOv8, ByteTrack, deep learning

## Abstract

In recent years, the application of deep learning technology in the field of food engineering has developed rapidly. As an essential food raw material and processing target, the number of kernels per maize plant is a critical indicator for assessing crop growth and predicting yield. To address the challenges of frequent target ID switching, high falling speed, and the limited accuracy of traditional methods in practical production scenarios for maize kernel falling count, this study designs and implements a real-time kernel falling counting system based on a Convolutional Neural Network (CNN). The system captures dynamic video streams of kernel falling using a high-speed camera and innovatively integrates the YOLOv8 object detection framework with the ByteTrack multi-object tracking algorithm to establish an efficient and accurate kernel trajectory tracking and counting model. Experimental results demonstrate that the system achieves a tracking and counting accuracy of up to 99% under complex falling conditions, effectively overcoming counting errors caused by high-speed motion and object occlusion, and significantly enhancing robustness. This system combines high intelligence with precision, providing reliable technical support for automated quality monitoring and yield estimation in food processing production lines, and holds substantial application value and prospects for widespread adoption.

## 1. Introduction

Maize, as one of the world’s three major food crops, nourishes approximately one-fifth of the global population and serves as a staple food for hundreds of millions of people. At the same time, the industrial significance of the maize crop is unmatched when compared to other cereal crops, and it is used as a raw material for over 3000 products in various sectors, namely, sweeteners, cosmetics, textiles, gum, alcoholic beverages, films, packaging, and paper industries [1]. Its yield and utilization profoundly influence global food security, economic development, and even the energy landscape. Maize kernels, serving as the primary storage site of nutrients in maize, have demonstrated certain preventive and therapeutic effects against conditions such as coronary heart disease, atherosclerosis, hyperlipidemia, and hypertension [2], and can also be directly used as feed for poultry and other livestock [3]. The number of maize kernels serves as a more accurate indicator for assessing crop growth and yield potential, providing scientific evidence and decision-making support for farmers and agricultural managers. Furthermore, it enables breeders to identify differences in kernel numbers among various varieties, thereby offering a foundation for breeding improvement.

From a traditional perspective, radar technology has achieved significant development and application in smart agriculture [4,5]. Numerous researchers have conducted extensive studies on maize, ranging from plant phenotypic measurement [6] and remote sensing imaging [7] to agricultural by-product processing [8] using various engineering techniques. Liu et al. developed an accurate and effective method for determining maize leaf azimuth and plant spacing using Light Detection and Ranging (LiDAR) technology. They collected three-dimensional point cloud data of maize plants and achieved effective 3D morphological reconstruction through multi-frame stitching, with R^2^ values of 0.87 and 0.83 for leaf azimuth and interplant spacing detection, respectively [9]. Gu et al. utilized UAV-based LiDAR technology to quantitatively analyze the impact of different growth stages and lodging severities on the self-recovery ability of maize plants. They validated the accuracy of UAV-LiDAR point cloud data in predicting the plant height and lodging angle of lodged maize and further examined how the self-recovery capacity of maize plants is manifested across various growth stages and levels of lodging severity [10]. Yadav et al. proposed a study on detecting VC plants in maize fields using RGB images collected by unmanned aerial vehicles (UAVs), aiming to maximize the true positive detection of VC plants in maize fields while minimizing the infestation of boll weevil pests. Su et al. [11] combined ground-based radar with a designed automatic extraction algorithm to identify points on maize leaves from large, unstructured LiDAR point cloud data. The results showed that the final accuracy could reach 94.1% [12]. Most of the researchers in the aforementioned studies adopted radar technology, using radar equipment to collect data and construct models of maize, ultimately achieving the goal of analyzing maize traits. However, the application of LiDAR in agriculture also has certain drawbacks. The point cloud data generated by LiDAR are massive and require high-performance computing platforms for processing. In addition, LiDAR faces difficulties in classifying crop types and characteristics, necessitating assistance from visible light imaging.

In recent years, computer vision technology has experienced rapid development in the field of smart agriculture [13,14]. Deep learning, as an advanced artificial intelligence technology, has already found extensive applications in the field of agricultural engineering [15,16], achieving significant progress in areas such as weed identification and management [17], crop yield prediction [18], and pest and disease monitoring [19]. In recent years, numerous researchers have combined deep learning techniques with various types of data in the field of agricultural engineering to achieve more accurate monitoring of crop growth conditions and food classification, thereby providing better decision-making support for professionals in agricultural engineering [20]. In the field of maize research, BT Kitano et al. employed a low-cost unmanned aerial vehicle (UAV) platform to capture images of maize fields and applied deep learning techniques to count maize plants, ultimately achieving automation of this process and reducing the need for manual labor [21].

Yang et al. proposed a maize variety recognition model based on a Convolutional Neural Network (LeNet-5) combined with near-infrared (NIR) spectroscopy and deep learning techniques, enabling efficient and rapid identification of maize varieties. The model achieved an accuracy of 99.20%, offering a new approach for maize variety classification [22]. Amin et al. developed an end-to-end deep learning model to distinguish between healthy and unhealthy maize leaves. The model leverages two pre-trained Convolutional Neural Networks (CNNs), EfficientNet-B0 and DenseNet-121, to extract deep features from maize plant images, achieving a classification accuracy of 98.56% [23]. Divyanth et al. developed a novel two-stage approach based on deep learning, employing the SegNet, U-Net, and DeepLabV3+ architectures to train three semantic segmentation models for each stage of maize disease. This method lays the foundation for developing field-ready disease management systems [24]. Xiao et al. mounted RGB and MicaSense multispectral cameras on UAVs to collect images of maize fields and utilized YOLOv5 to count maize plants. They demonstrated the feasibility of using the Otsu thresholding method to automatically extract plant height, NDVI, and NDRE values. By analyzing different maize field management practices, they verified that these variations significantly affect the emergence rate and concluded that fertilizing near the seeds is the most effective method for achieving higher emergence rates in experimental fields [25].

However, despite the significant potential demonstrated by deep learning techniques in maize-related research fields, there exists a notable deficiency in specialized studies focusing on maize kernels. On one hand, existing achievements are mostly concentrated on macroscopic aspects such as maize plant phenotype analysis, variety identification, or disease detection, with a limited number of in-depth studies specifically centered on maize kernels. On the other hand, even when kernel-related content is involved, it fails to fully integrate with the actual working conditions of food industrial production. In industrial assembly lines, maize kernels are often in a state of dynamic falling. During their movement, random flipping of directions and rapid changes in spatial positions (such as mutual occlusion and trajectory crossing) can greatly interfere with the accuracy of traditional recognition algorithms. Meanwhile, these factors lead to problems such as target loss and repeated counting in the process of kernel tracking, seriously affecting the counting efficiency and result reliability.

This technological gap poses numerous challenges in real-world production scenarios. For food processing enterprises, relying on manual counting is not only time-consuming and labor-intensive but also prone to errors caused by operator fatigue, leading to high costs and low reliability. Moreover, existing counting methods developed for static or semi-static conditions are poorly suited to the dynamic scenario of high-speed falling kernels, making it difficult to meet the dual requirements of counting speed and accuracy in industrial production. Therefore, the development of a technical solution capable of accurately identifying the diverse morphological states of maize kernels during free fall—such as side-rolling, overlapping, and rotation—while continuously tracking their dynamic trajectories and performing precise counting, is crucial to addressing the challenge of quantitative kernel detection in the food industry.

Based on this, this study proposes a purpose-built integrated counting hardware system that innovatively combines the dynamic capture capabilities of a high-speed camera with deep learning techniques. The YOLOv8 algorithm is employed to achieve real-time detection of kernels in high-speed motion, while the ByteTrack tracking algorithm is simultaneously introduced to continuously track the motion trajectories of the kernels, ensuring that the kernels can be efficiently identified and accurately counted throughout the entire falling process within the field of view of the high-speed camera. This technical solution not only fills the research gap in batch counting of maize kernels under dynamic conditions, but its core concept and technical framework can also be extended to detection scenarios involving other grain kernels such as wheat, rice, beans, etc. It provides a reusable technical paradigm for quantitative analysis of kernels in the entire food production process and has important practical significance for improving the level of automated detection in the food industry, reducing production costs, and ensuring product quality.

## 2. Materials and Methods

### 2.1. Counting Device System Design

#### 2.1.1. Hardware Components of the Counting System

**Figure 1:**
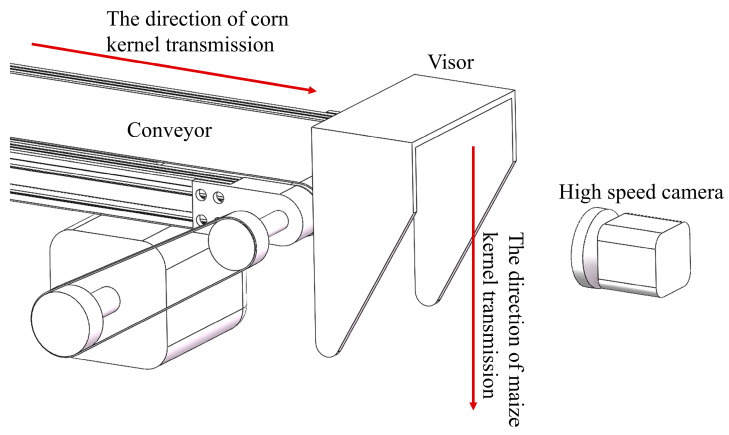
Schematic diagram of system structure.

The hardware structure of the counting device system is shown in Figure 1 and primarily consists of a conveyor belt, a high-speed camera, a light source, and a light-shielding board. The high-speed camera, serving as the visual acquisition module. When maize kernels enter the image acquisition area of the high-speed camera, they are captured and subsequently counted. In a machine vision system, the high-speed camera plays a critical role. Compared to a conventional camera, its notable feature lies in its acquisition rate of no less than 1000 frames per second. This capability effectively extends the temporal resolution of the maize kernel falling process, thereby significantly enhancing the feasibility of tracking and counting moving kernels with subsequent algorithms. In this system, a Chronos 2.1-HD high-speed camera(high-speed camera from Krontech in Canada) is employed.

#### 2.1.2. Principle and Workflow of the Counting Device

**Figure 2:**
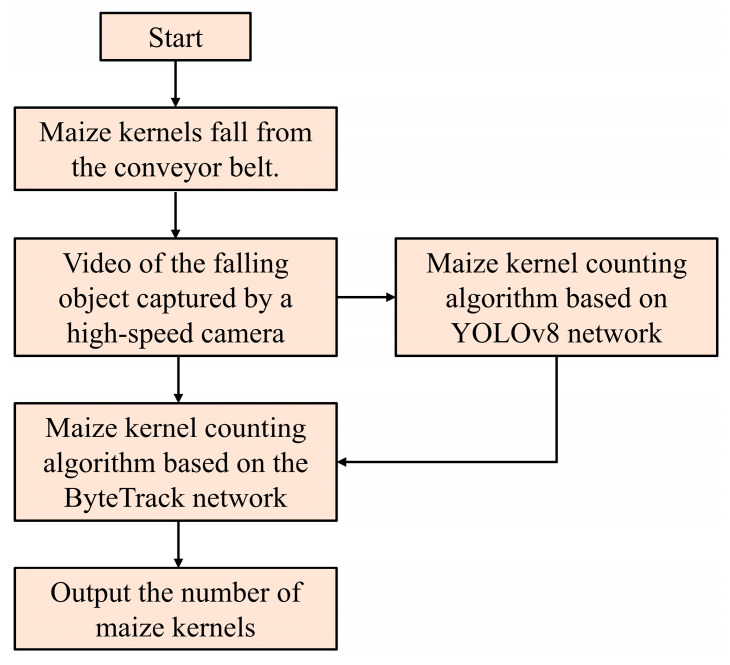
System workflow diagram.

The working principle of the counting device system is illustrated in Figure 2. It simulates the process of kernels falling from the conveyor belt during crop production. The conveyor operates at a speed of 25 revolutions per minute. To prevent interference from ambient light on image acquisition, the acquisition module employs an enclosed light-shielding board and fixed illumination. Maize kernels naturally fall from the conveyor into the field of view of the high-speed camera, which captures video of the falling process. The recorded video is transmitted to a computer, where a deployed ByteTrack network processes the footage to count the falling maize kernels.

#### 2.1.3. Experimental Data Processing Block

The experimental data processing module serves as the core component for achieving precise maize kernel counting, with its workflow centered on dynamic data acquisition, recognition, and tracking throughout the kernel falling process.

First, data acquisition of falling maize kernels is carried out using the constructed hardware system. Under simulated food production line conditions, maize kernels naturally fall from the operating conveyor belt. A high-speed camera (Chronos 2.1-HD) is positioned within a stable illumination environment created by light-shielding panels and fixed light sources, capturing the dynamic falling process at a frame rate of no less than 1000 frames per second. This process ensures high temporal resolution recording of high-speed moving targets, providing clear and continuous raw data for subsequent processing.

Subsequently, the video stream data of falling kernels are extracted. The raw data collected by the high-speed camera are transmitted to the computer in the form of video streams. The system conducts preprocessing on the video streams, including frame sequence extraction, image denoising, and contrast enhancement. By temporally segmenting the continuous video stream into an ordered sequence of static frames, the dynamic falling process is transformed into analyzable static image sequences frame by frame, providing standardized input data for the target recognition algorithm.

Next, maize kernel recognition based on the YOLOv8 model is carried out. The preprocessed image sequences are input into the trained YOLOv8 target detection model. Through deep convolutional neural network learning, the model extracts morphological features of maize kernels, such as contour, texture, and size, to localize and classify kernels within each frame, outputting the corresponding bounding box coordinates and confidence scores. The high real-time performance and detection accuracy of YOLOv8 ensure efficient target recognition even when kernels are moving rapidly or partially occluded, laying a foundation for subsequent tracking.

**Figure 3:**
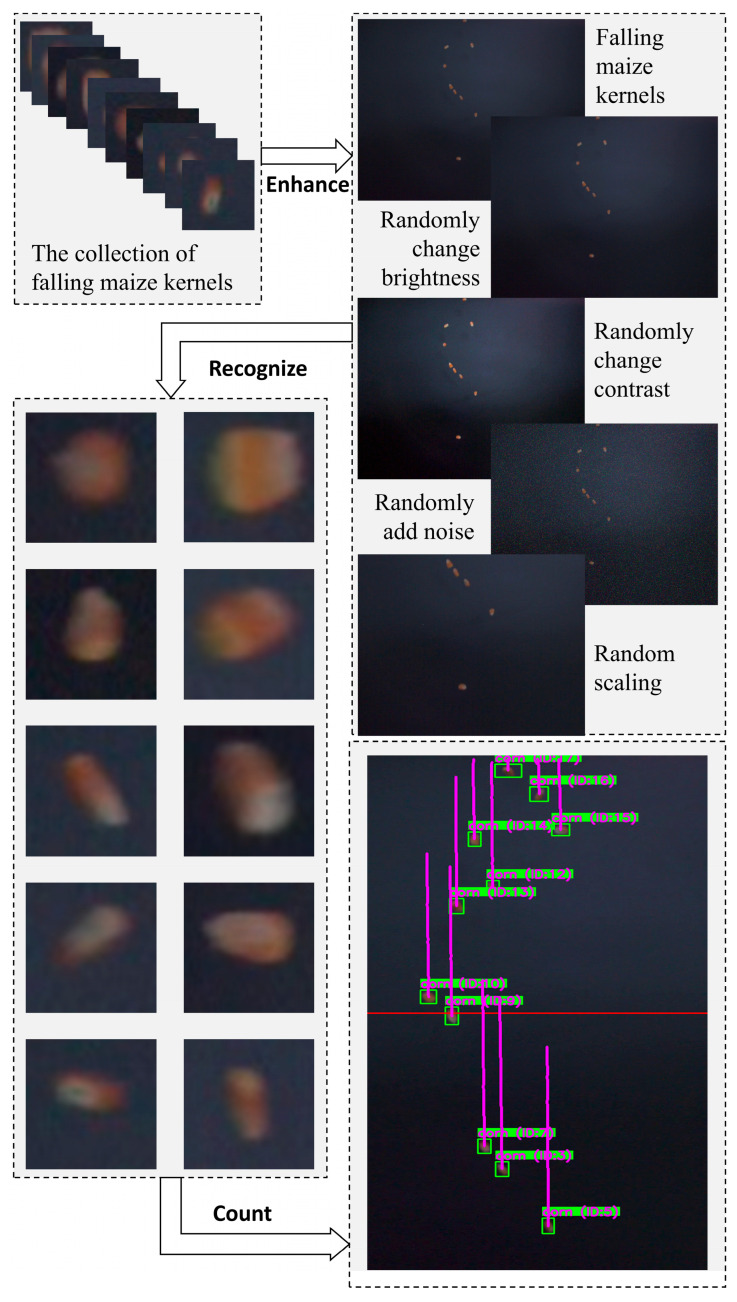
Data processing block diagram.

Finally, kernel target tracking and counting line detection are accomplished based on the ByteTrack technique. To address the issue of discontinuous target IDs in single-frame recognition results, the system introduces the ByteTrack multi-object tracking algorithm. This algorithm associates the position, motion trajectory, and appearance features of kernels across adjacent frames, assigning each kernel a unique and continuous ID to achieve cross-frame trajectory tracking. Meanwhile, a virtual counting line is set in the video stream (e.g., a specific position on the kernel falling path). When the tracking trajectory crosses the counting line and the target confidence exceeds the predefined threshold, the system records a valid count, and the total number of kernels in the batch is finally obtained by accumulation. Through the integration of YOLOv8 and ByteTrack, the system effectively resolves the ID switching problem caused by high-speed movement and occlusion of kernels, ensuring the accuracy and robustness of counting results. The experimental data processing module is illustrated in Figure 3.

### 2.2. Experimental Data Collection

**Figure 4:**
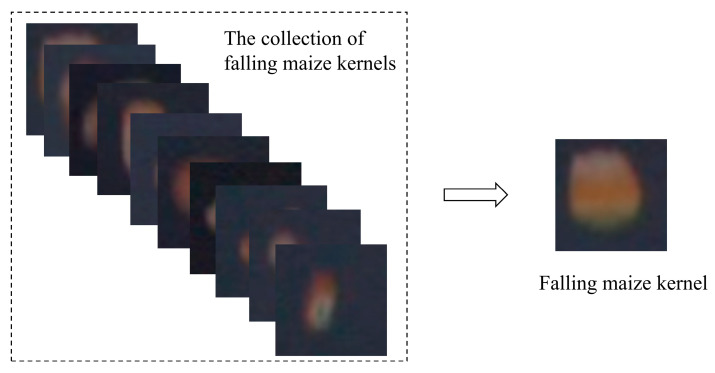
Schematic diagram of the maize kernel dataset.

The experimental material consisted of maize kernels, and video datasets capturing the falling process of the kernels were obtained using a high-speed camera. Due to uncertainties in the natural environmental background and lighting conditions, a uniform background panel and fixed light source were employed during data acquisition. The video datasets were extracted frame by frame into image datasets. Given that the kernel features exhibited minimal differences between consecutive frames during high-speed filming, one image was extracted every 80 frames to constitute the dataset. The resulting dataset encompasses various kernel postures during the falling process. A representative example of the dataset is shown in Figure 4.

Using Labelme 5.2.1 software, maize kernels in the falling process were annotated to obtain TXT files containing the kernel center coordinates as well as the annotated width and height information. The maize kernel dataset was then divided into training and validation sets, with the data split detailed in Table 1.

**Figure 5:**
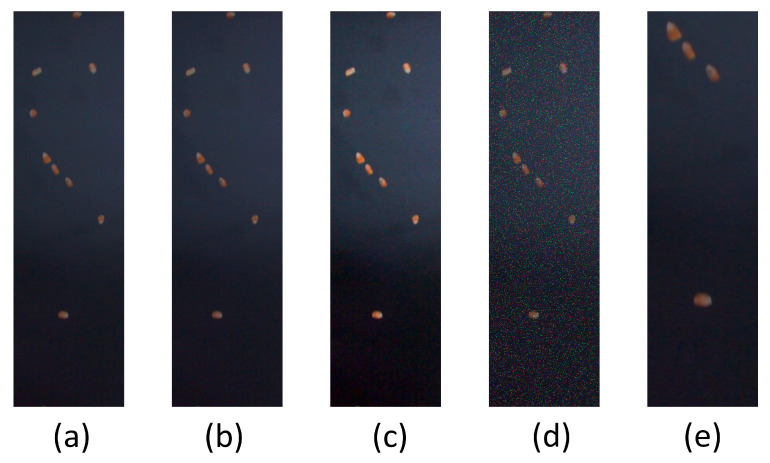
Data augmentation effect image of falling maize kernels. (**a**) Falling maize kernels; (**b**) randomly change brightness; (**c**) randomly change contrast; (**d**) randomly add noise; (**e**) random scaling.

Data augmentation was performed on both the original dataset and preprocessed static images to enhance the algorithm’s generalization ability and increase sample diversity. Specifically, random brightness and contrast adjustments enable the algorithm to adapt to illumination variations across different acquisition environments, ensuring robustness under varying light intensities. Random noise addition simulates interference during image acquisition and transmission, ensuring stable performance in the presence of noise. Random scaling facilitates effective recognition of targets of varying sizes. Collectively, these strategies significantly enrich the diversity of the training dataset, reduce the risk of overfitting, and improve the algorithm’s generalization to unseen data, as illustrated in Figure 5 with the augmentation effect of falling maize kernels.

Notably, common augmentation techniques such as flipping and rotation were not employed in this study. This decision was based on the observation that the original dataset already encompasses diverse morphologies and angles of maize kernels captured during their falling process. The application of flipping or rotation would potentially introduce unnatural morphological characteristics inconsistent with the actual physical properties of maize kernels, thereby rendering such augmentation methods unsuitable for the current dataset.

### 2.3. Crop Kernel Counting and Tracking Method

#### 2.3.1. Counting Method

In this study, a counting method for maize kernels was constructed based on high–speed imaging technology, aiming to achieve accurate counting of maize kernels through scientifically sound experimental design and technical procedures.

The experimental materials selected were mature maize kernels, and the selection process emphasized uniformity. This is because uniformly selected mature maize kernels have relatively consistent physical properties such as size, shape, and density, which can reduce the interference caused by differences in kernel characteristics during the counting process and lay a good foundation for the accuracy of subsequent counting results.

**Figure 6:**
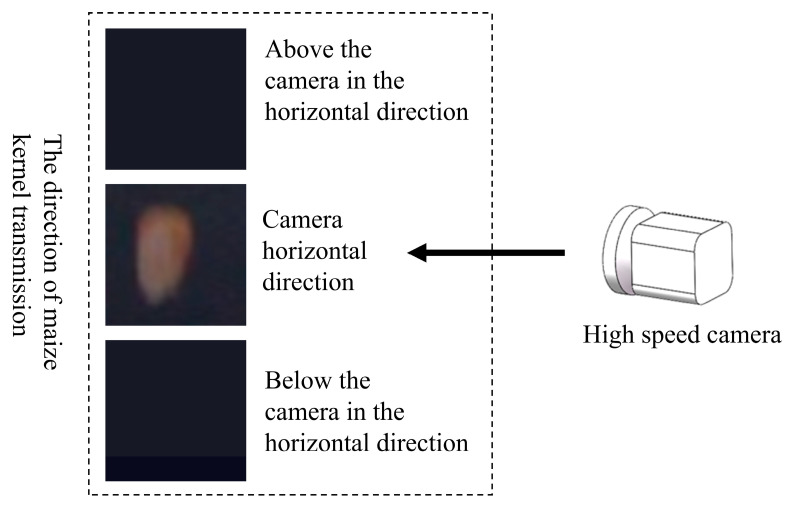
High-speed camera captures data of maize kernels falling from high to low.

During the experiment, the maize kernels were released sequentially from above the horizontal plane, the horizontal plane, and below the horizontal plane in that order, as shown in Figure 6. This multi-directional release approach was designed to simulate various motion states of maize kernels in real-world scenarios as closely as possible. By covering various possible movement trajectories, it can ensure that the collected data is more comprehensive and representative, avoiding the potential bias introduced by one-directional release.

To ensure the quality of image acquisition, a high-speed camera was installed horizontally above the experimental area. Additionally, a monochromatic background panel and a fixed light source were employed in the experiment. The monochromatic background effectively reduces the complexity of the image background, making the maize kernels more prominent and facilitating subsequent image processing and recognition. The fixed light source plays a critical role in eliminating interference from environmental background and light fluctuations. Natural light is typically unstable, with variations in intensity and direction due to factors such as weather and time, which can negatively affect the clarity and consistency of the captured images. The fixed light source provides a stable and uniform lighting environment, ensuring that images of maize kernels captured at different times and locations maintain consistent brightness and contrast, thereby enhancing the reliability of the image data.

**Figure 7:**
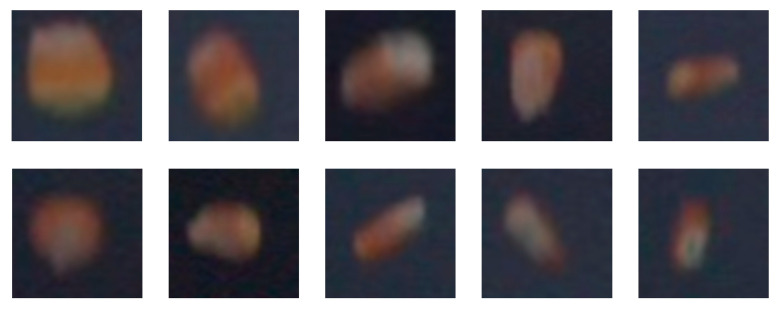
Data captured by high-speed cameras when different maize kernels fall.

The image acquisition system was employed to record the motion trajectories of the maize kernels in each direction. During the falling process of the maize kernels, the high–speed camera continuously captured images, and the image acquisition system integrates these images into a video dataset. This video dataset contains rich information about the motion of maize kernels, including their positions, velocities, and directions at different time points. The data captured by the high-speed camera during the falling process of maize kernels is shown in Figure 7.

Finally, accurate counting of maize kernels was achieved based on the video sequences. By analyzing the video sequences, the motion trajectories of each maize kernel could be tracked and identified. Through appropriate algorithmic processing, each individual kernel in the video could be distinguished, and the total number of maize kernels was accurately calculated. This method integrates high-speed imaging technology with image processing techniques, enabling efficient and accurate counting of maize kernels.

#### 2.3.2. Line-Crossing Counting Method

When using the ByteTrack algorithm to track maize kernels, frequent ID switches can occur, especially as kernels rotate while entering the camera’s field of view. Such ID instability may lead to overcounting if the kernel count is directly based on ID information. Consequently, relying solely on ByteTrack-generated kernel IDs for total count results in significant errors. To address the issue of ID switching, a line-crossing counting method was designed.

**Figure 8:**
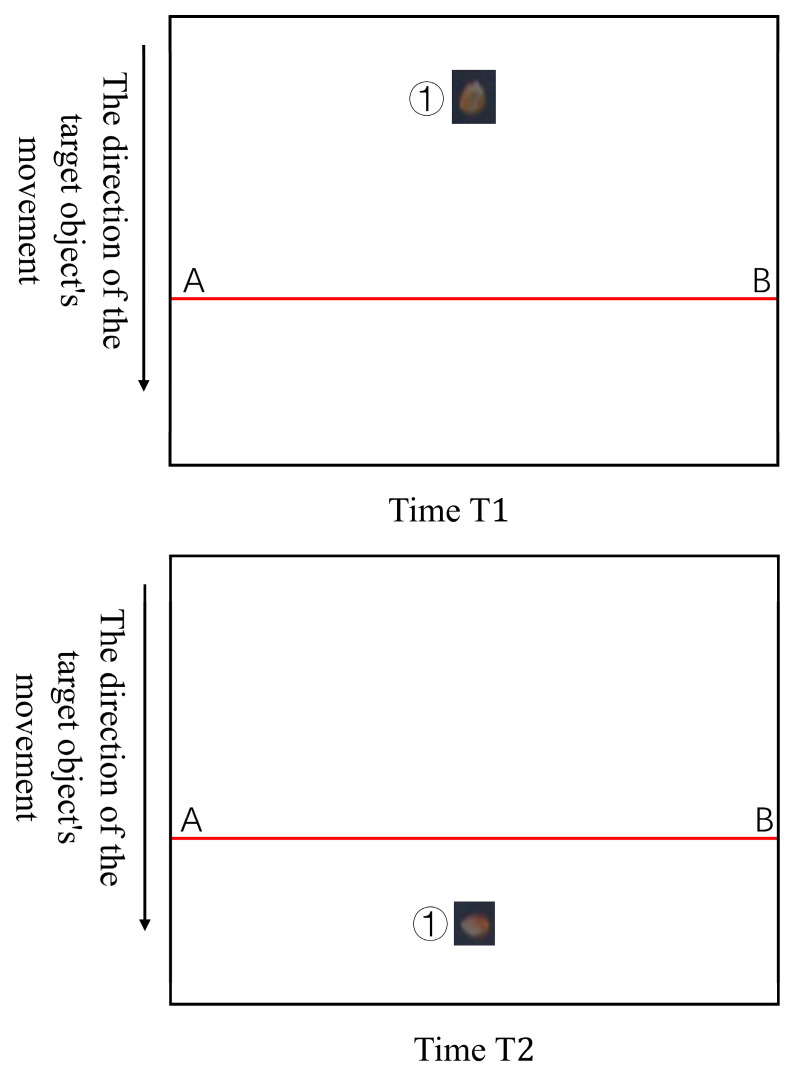
Line-crossing counting method.

As shown in Figure 8, point ① represents a maize kernel with the same ID, whose center coordinates are (x_1_, y_1_). The red line denotes the counting detection line, where point A has coordinates (x_a_, y_0_) and point B has coordinates (x_b_, y_0_). In this study’s counting model, a maize kernel with a given ID is only counted when, at time T_1_, its y_1_ coordinate is less than y_0_, and at a later time T_2_, y_1_ becomes greater than y_0_. The line-crossing counting method thus registers the kernel’s count upon crossing this detection line, effectively reducing duplicate counting of kernels during the counting process.

#### 2.3.3. YOLOv8 Model

YOLOv8, developed by Ultralytics and officially released in January 2023, is a state-of-the-art (SOTA) model that inherits the advantages of the YOLO series while introducing new features and improvements. Compared to its predecessor YOLOv5, the main changes include: replacing the C3 module with the C2f module in the backbone network; removing convolution operations during the upsampling process; and adopting a decoupled head structure (decoupled head) that separates classification and detection tasks, further reducing model complexity.

**Figure 9:**
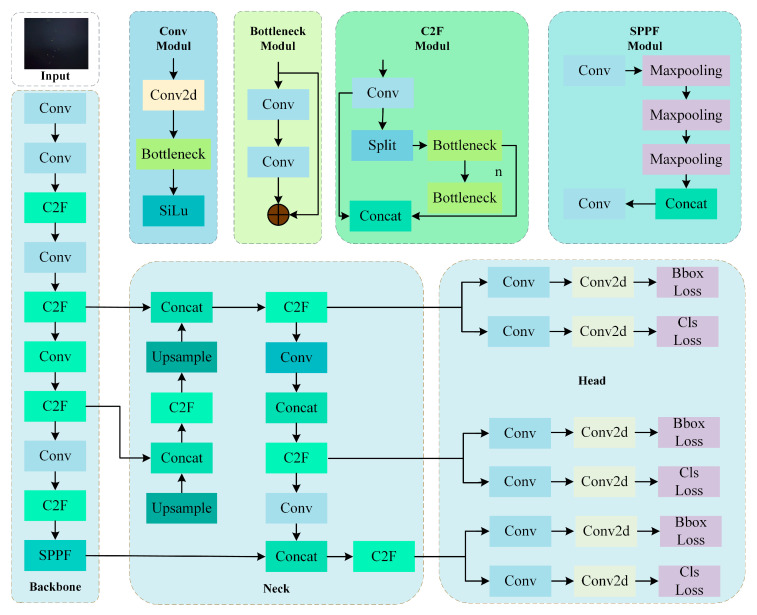
YOLOv8 model drawing.

The YOLOv8 network architecture consists of three components: the Input, Backbone, and Head. The input module feeds chestnut fruit images enhanced by Mosaic augmentation into the network. The C2f module comprises n Bottleneck layers, three convolutional layers, a Split operation, and multiple skip connections. The SPPF (Spatial Pyramid Pooling-Fast) module extracts multi-scale features through pooling operations with different kernel sizes, which are then aggregated and fused. The YOLOv8 model architecture is illustrated in Figure 9.

#### 2.3.4. ByteTrack Model

ByteTrack, proposed by Zhang et al. [26], is an innovative multi-object tracking (MOT) framework based on a detection-tracking paradigm. Its core contribution lies in introducing a hierarchical data association strategy that significantly enhances tracking robustness in complex dynamic scenes. Unlike traditional methods that rely solely on high-confidence detection boxes for association, ByteTrack systematically demonstrates the critical role of low-confidence detection boxes in maintaining trajectory continuity. The approach first predicts target states using a Kalman filter and employs Intersection over Union (IoU) or motion similarity metrics to perform an initial association between high-confidence detection boxes and existing trajectories. Subsequently, it innovatively introduces a secondary association mechanism that recovers unmatched high-confidence boxes from the initial matching and incorporates low-confidence detections, typically discarded in conventional processes, to associate with remaining unmatched trajectories. This staged processing effectively mitigates missed detections caused by occlusion, motion blur, or sudden scale changes, substantially reducing trajectory fragmentation and identity switches (IDS). Experiments on authoritative benchmarks such as MOT17 and MOT20 demonstrate that ByteTrack achieves outstanding performance—e.g., a 63.1% MOTA on the MOT17 test set—without relying on complex appearance models or additional optimization strategies, while maintaining efficient inference speed (≥30 FPS). Due to its excellent tracking capability, ByteTrack has recently been applied by some researchers in the field of agricultural engineering [27].

In the maize kernel counting system, recognition and tracking of falling kernels are achieved by integrating the YOLOv8 object detection framework with the ByteTrack multi-object tracking algorithm, forming a complete processing pipeline for dynamic falling scenarios.

The system first employs a high-speed camera to capture a continuous video stream of falling maize kernels, providing real-time image data support for subsequent recognition and labeling. For each frame of the video stream, the YOLOv8 object detection framework is applied to complete the recognition task of maize kernels. This framework can quickly and accurately locate the position information of maize kernels in the image, and separate them from the complex background through bounding boxes, achieving initial detection of maize kernels.

Building on the detection results, the ByteTrack multi-target tracking algorithm is used to continuously label and track the detected maize kernels. ByteTrack adopts a hierarchical data association strategy, first using Kalman filtering to predict the motion state of labeled maize kernels, and then using Intersection over Union (IoU) or motion similarity measurement to preliminarily associate the high-confidence bounding boxes detected by YOLOv8 in the current frame with the existing maize kernel trajectories, assigning a unique identity (ID) to each maize kernel to ensure its consistency in consecutive frames. For high-confidence bounding boxes that have not been successfully associated initially, as well as low confidence bounding boxes that are easily overlooked in conventional processing, ByteTrack introduces a secondary association mechanism to perform restorative matching with the remaining unmatched trajectories, effectively compensating for the temporary missed detections caused by high-speed grain movement, mutual occlusion, or motion blur, maintaining trajectory continuity, and reducing the occurrence of identity switching.

**Figure 10:**
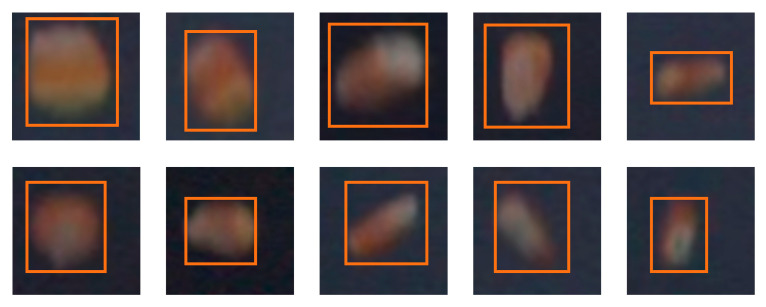
Mark the falling maize kernels.

Through the above methods, the system can stably identify and continuously label each maize kernel during the falling process, clearly record its motion trajectory in the video stream, and provide reliable basic data for subsequent counting and related analysis. The schematic diagram of the maize kernel dataset, shown in Figure 10, illustrates the labeled maize kernel targets clearly.

## 3. Results and Analysis

### 3.1. Model Evaluation Metrics

Recall, precision, and mean average precision (mAP) were employed as evaluation metrics for the maize kernel object detection algorithm. Recall refers to the probability that correctly labeled samples are predicted accurately, where TP (True Positives) denotes the number of correctly predicted positive samples, and FN (False Negatives) represents the number of positive samples incorrectly predicted as negative. Precision is defined as the ratio of correctly predicted positive samples to the total number of predicted positive samples, with FP (False Positives) indicating the number of negative samples incorrectly predicted as positive. Additionally, mean average precision (mAP) is a commonly used metric in object detection models, representing the average precision across all classes.

(1) $$\mathrm{Recall}=\frac{TP}{TP+FN}$$

(2) $$\mathrm{Precision}=\frac{TP}{TP+FP}$$

(3) $$AP=\int_{0}^{1}Precision(r)dr$$

(4) $$mAP=\frac{1}{N}\sum_{i=1}^{N}AP_{i}$$

Despite the rapid advancement of various algorithms in the current market, YOLOv8 has demonstrated strong performance and distinct advantages for our specific application scenario. The results presented in Table 2 reveal that while YOLOv8 exhibits excellent performance, its outcomes are marginally different from those of YOLOv5. Specifically, YOLOv5 achieved a Precision of 0.997, Recall of 0.995, and mAP of 0.998, whereas YOLOv8 obtained a Precision of 0.998, Recall of 0.996, and mAP of 0.998. Following a result-oriented principle, we ultimately selected YOLOv8 as it can adequately fulfill the requirements of our application scenario. Adhering to a results-oriented principle, we ultimately chose YOLOv8 because it not only fully satisfies the requirements of our application scenarios but also provides a more reliable performance in practical deployment due to its higher accuracy and lower false alarm rate. In addition, we also validated the latest YOLOv11 model on our dataset, which demonstrated competitive performance but did not exhibit a substantial improvement compared to YOLOv8. Given that YOLOv8′s ecosystem is more mature and capable of sufficiently accomplishing our target tasks, we have ultimately made this choice.

### 3.2. Maize Kernel Object Detection

The maize kernel object detection algorithm, based on the YOLOv8 network, was trained on a personal computer configured with a 12th Gen Intel^®^ Core^TM^ i7-12700H CPU, an NVIDIA GeForce RTX 3060 Laptop GPU, 16 GB of RAM, Python 3.8, and PyTorch 2.0.0+cu118. The training parameters for the YOLOv8 model were set as follows: input image size of 640 × 640 pixels, batch size of 16, a maximum of 300 iterations, a momentum factor of 0.9, a weight decay coefficient of 0.0005, and an initial learning rate of 0.01.

**Figure 11:**
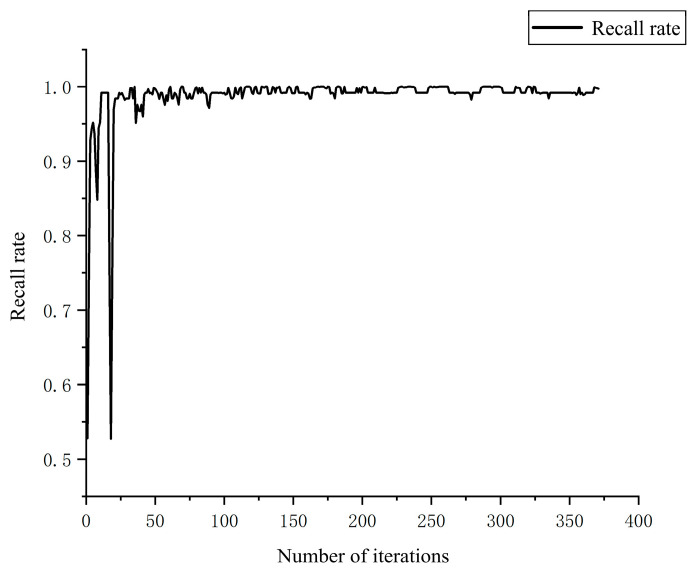
Recall Rate Chart.

**Figure 12:**
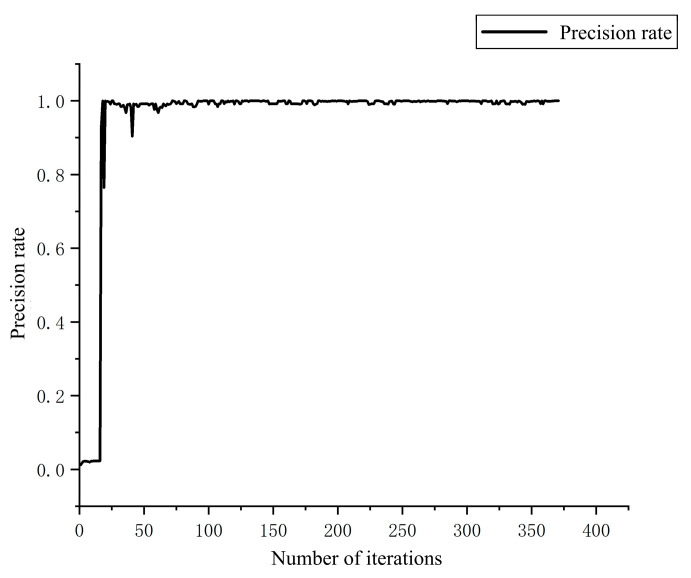
Precision Rate Chart.

**Figure 13:**
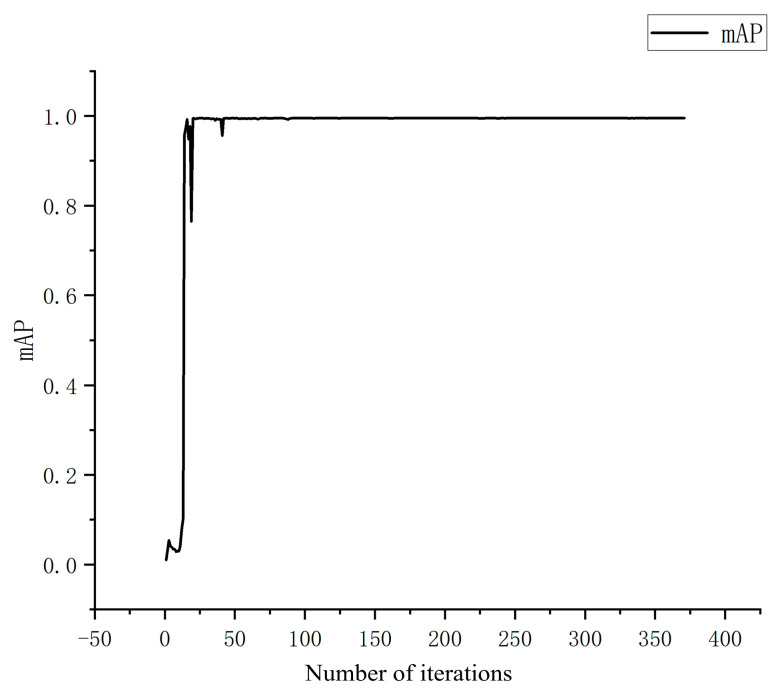
mAP chart.

Using the training set composed of the aforementioned images, maize kernels were detected. The resulting recall and precision rates are shown in the figure. After 300 iterations, the model converged, achieving a recall rate of 0.99, indicating that the model can accurately detect maize kernels during the falling process. Figure 11, Figure 12 and Figure 13 illustrate the changes in precision, recall, and mAP with respect to the number of iterations.

### 3.3. Maize Kernel Counting Results

**Figure 14:**
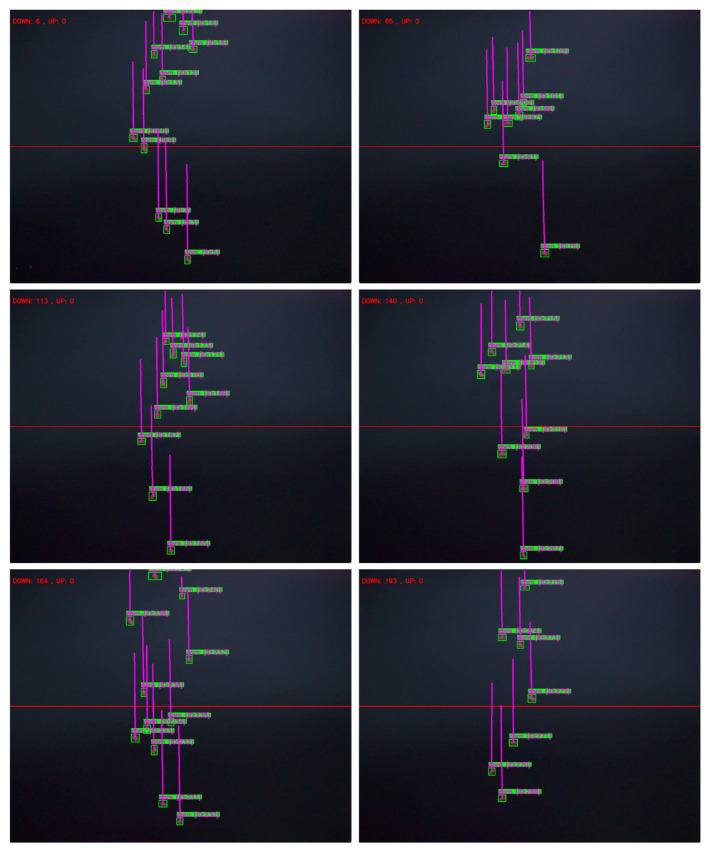
ByteTrack Counts maize Kernels.

In the manually generated video dataset, 300 kernels were used. These kernels were allowed to free-fall from the conveyor belt into the field of view of the high-speed camera, where the ByteTrack algorithm was applied for object counting. As shown in Figure 14, once a maize kernel enters the camera’s field of view, it is identified as a green target. When the kernel crosses the red line, it is counted once. Table 3 presents the statistical results of the experiments. Based on five test trials, the counting accuracy in each trial exceeded 99%, indicating that the model can accurately track and count maize kernels during the falling process.

## 4. Conclusions

The maize kernel batch counting system developed in this study, based on the YOLOv8-ByteTrack framework, achieved a counting accuracy exceeding 99% in dynamic falling scenarios. This result not only validates the effectiveness of the proposed technical approach but also highlights the synergistic advantages of deep learning and multi-object tracking (MOT) technologies in agricultural dynamic counting applications. From a technical perspective, the C2f module in YOLOv8 employs a multi-branch feature fusion architecture that enhances the model’s ability to extract discriminative features from kernels exhibiting complex deformations and rotations during high-speed motion, thereby ensuring robust detection even under rapid movement conditions. Meanwhile, ByteTrack introduces an innovative strategy that leverages low-confidence detection bounding boxes for secondary association, effectively mitigating trajectory fragmentation issues commonly caused by occlusions or motion blur in conventional tracking algorithms.

A critical innovation of this study lies in the proposed line-crossing counting method, which incorporates spatiotemporal coordinate constraints—specifically, requiring that the same object ID be detected on opposite sides of the counting line at different time instances. This design fundamentally prevents duplicate counting caused by ID switching, keeping the cumulative counting error below 0.7%. This performance represents a significant improvement over traditional methods that rely solely on ID-based statistics and offers a novel logical validation mechanism for dynamic object counting tasks.

Compared with existing studies, the proposed system demonstrates significant innovation in both technical approach and application scenarios. In contrast to the ground-based radar method adopted by Su et al. [12], which achieved a leaf extraction accuracy of 94.1%, the high-speed vision approach in this study requires only 1/20 of the data volume of point cloud methods and does not rely on high-performance computing platforms, making it more suitable for real-time demands on production lines. Compared with the static plant counting method based on unmanned aerial vehicles (UAVs) proposed by Kitano et al. [21], the present system targets the dynamic process of kernel free-fall and addresses motion blur through the use of high-speed cameras operating at 1000 fps, thereby filling a technological gap in dynamic kernel counting during food processing. While the maize variety recognition model developed by Yang et al. [22] achieved a high accuracy of 99.20% in static classification, it lacks the capacity for continuous tracking of kernels in dynamic bulk conditions. This study integrates detection, tracking, and counting into a unified pipeline, thereby extending the application of deep learning in agriculture from single-object recognition to dynamic quantitative analysis for the first time. Regarding the counting error reported by Xiao et al. [25], in maize plant counting, the main issue lies in the absence of a trajectory validation mechanism for moving targets. This deficiency is effectively addressed in the present study through the proposed line-crossing algorithm.

The outcomes of this study hold significant value in both academic and industrial domains. Academically, it establishes a comprehensive technical framework for dynamic kernel counting and validates the applicability of multi-object tracking (MOT) algorithms within agricultural engineering. Notably, the line-crossing counting method proposed herein further expands the scope of MOT applications. From an industrial perspective, the system can be directly integrated into maize processing lines to replace conventional manual sampling methods, achieving a substantial enhancement in counting efficiency. It also provides precise quantification of kernel numbers per plant, which is crucial for breeding research. Furthermore, the proposed technical solution is readily extendable to the counting of other grain types such as soybeans and wheat, offering a valuable reference for automation upgrades in agricultural product processing and feed production sectors.

Nevertheless, this study has certain limitations. First, the dataset includes only a single maize kernel variety and does not encompass kernels from different maize types with varying sizes and shapes (e.g., sweet maize and field maize), which may affect the generalization capability of the model. To address this, future work should expand the dataset to include multiple maize varieties with diverse morphological characteristics. These will include sweet maize with larger and plumper kernels, field maize with smaller and more compact shapes, and kernels with natural variations such as broken or misshapen ones. This expansion will enable the model to learn more comprehensive feature representations, enhancing its adaptability to different production scenarios. Second, the kernel falling density in the experiments was controlled at 5–10 kernels per frame (low-density scenario), where the system achieved a counting accuracy of over 99%, benefiting from YOLOv8’s robust detection of overlapping targets (up to 30% occlusion) and ByteTrack’s effective trajectory association. However, the counting performance under high-density conditions (>20 kernels per frame) was not evaluated; in such scenarios, severe mutual occlusion (occlusion rate exceeding 50%) and trajectory cross may lead to increased errors, with counting errors mainly caused by detection failure due to blurred frames from high-speed movement of kernels and tracking losses due to the severe overlapping of multiple kernels. The current model may struggle to distinguish individual kernels in highly cluttered motion states. Future research will optimize the association strategy of ByteTrack by incorporating appearance features (e.g., texture and color) to enhance target discrimination under high occlusion and further improve YOLOv8’s detection capability for overlapping kernels through multi-scale feature fusion enhancement, with the aim of maintaining an accuracy level above 95% in high-density scenarios. Third, the high cost of the Chronos 2.1-HD high-speed camera hinders the large-scale deployment of the system. Lastly, the current system achieves a processing speed of 30 FPS on a standard PC, which may not meet the demands of ultra-high-speed production lines. Future research can be improved in the following directions: expanding the dataset to include multiple kernel varieties and operational conditions to enhance model robustness; optimizing ByteTrack’s association strategy (e.g., incorporating appearance-based matching) to handle high-density occlusion; exploring frame-rate compensation algorithms using standard cameras to reduce hardware costs; and increasing processing speed to over 100 FPS through model lightweighting (e.g., using YOLOv8-nano) and GPU-based parallel acceleration.

From multiple perspectives of technological application, the deployment of this system can reduce manual intervention in food processing and alleviate visual fatigue caused by prolonged counting tasks, aligning with the human-centered development trend in intelligent manufacturing. From the viewpoint of food security, accurate kernel counting provides micro-level data support for yield estimation, contributing to the optimization of planting strategies and supply chain management. At the methodological level, the problem-driven, technology-integrated, and scenario-adapted approach validated in this study offers a valuable reference for addressing other agricultural engineering challenges, promoting the practical implementation of interdisciplinary technologies in real-world production environments.

## Figures and Tables

**Table 1:** Distribution table for maize kernel dataset.

Category	Number of Images	Number of Labels
Training Set	304	2432
Validation Set	72	584

**Table 2:** Statistical table of different models.

Model.	Precision	Recall	mAP
YOLOv5	0.997	0.995	0.998
YOLOv8	0.998	0.996	0.998
YOLOv11	0.998	0.997	0.998

**Table 3:** Statistical table of test results.

Trial No.	Ground Truth	Predicted Count	Accuracy
1	300	299	99.6%
2	300	300	100%
3	300	298	99.3%
4	300	299	99.6%
5	300	298	99.3%

## Data Availability

Data are contained within the article.
